# Neglected challenges in the control of animal rabies in China

**DOI:** 10.1016/j.onehlt.2021.100212

**Published:** 2021-01-05

**Authors:** Faming Miao, Nan Li, Jinjin Yang, Teng Chen, Ye Liu, Shoufeng Zhang, Rongliang Hu

**Affiliations:** Key Laboratory of Jilin Province for Zoonosis Prevention and Control, Institute of Military Veterinary Medicine, Academy of Military Medical Sciences, 666 Liuying West Road, Jingyue Economic Development Zone, Changchun 130122, Jilin Province, China

**Keywords:** Rabies, Animal, Epidemics, One health, Virus

## Abstract

Complex rabies transmission dynamics, including in dogs, wildlife livestock, and human-acquired rabies, can be observed in China. A temporary decrease in human rabies deaths with a simultaneous increase in animal rabies transmission is a typical example of “sectoral management separation” but not of the recommended “one-health” concept. In contrast to reliance on mass dog vaccination, reliance on postexposure prophylaxis to reduce human rabies burden is costly and ineffective in the prevention of rabies transmission from dogs to humans and other susceptible animal species. To answer the WHO call for the “elimination of dog-mediated human rabies by 2030,” China faces the challenge of a lack of a strong political commitment and a workable plan and must act now before the rabies transmission dynamics become increasingly complicated by spreading to other species, such as ferret badgers in the Southeast and raccoon dogs and foxes in the North.

## 1 Introduction

**Fig. 1:**
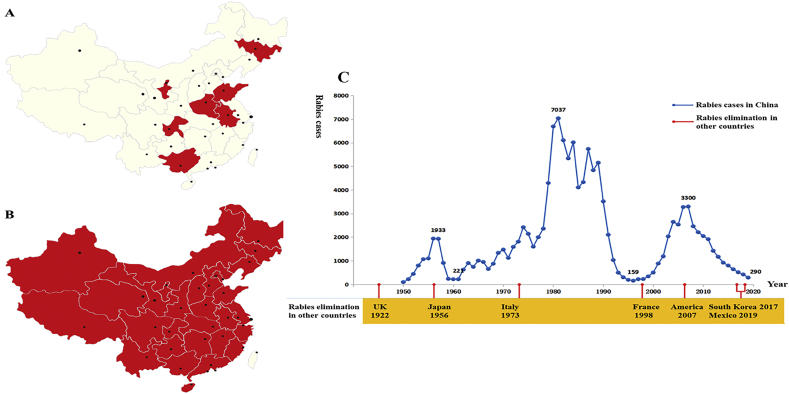
Spatial-temporal dynamics of human rabies in China. A) distribution of human rabies 1990–2000; B) distribution of human rabies 2010–2019; C) human rabies cases followed by chronological summation.

Rabies is one of the oldest zoonotic diseases worldwide caused by rabies virus (RABV) and rabies-related lyssaviruses in the genus *Lyssavirus* of the family *Rhabdoviridae* [1]. Rabid carnivores, especially dogs, are the main causes of human and domestic animal cases, commonly via bite injury. Therefore, human rabies elimination strictly depends on the control of rabies in host animals [1]. Dog-associated rabies in China is a continuous public health threat due to intra- and interspecific RABV infection among dogs, humans, domestic animals, and wildlife [2]. Human rabies deaths peaked with 3300 cases in 2007 and diminished to 290 in 2019, suggesting a current decline in the disease (Fig. 1C). However, the decline in human rabies deaths is mainly due to heavy postexposure prophylaxis (PEP) administrations and public PEP awareness [3]. Approximately 40 million people in the country are injured by dogs every year. However, medical professionals in China seldom practice rabies risk assessment, resulting in the administration of 12–15 million doses of human rabies vaccines to individuals bitten by any rabies-suspected animals or unknown animals [4,5]. The rabies epidemic has continuously expanded geographically over the entire country, and new cases have been recorded in previously rabies-free and low-incidence provinces, such as the Ningxia Hui Autonomous Region, Qinghai, Gansu, Beijing, Jilin, and Tibet, in the last decade [6] (Fig. 1A-B). This “phenomenon” of a temporary decrease in human rabies deaths and a simultaneous increase in animal rabies transmission is a typical example of “sectoral management separation” and not of the recommended “one-health” approach [2].

The decrease in human deaths is mistakenly labeled “progress” by the political sector, leading to the relaxation of subsequent control efforts. Intermittent “relaxation” and commitment leads to human rabies “peaks” or “epidemic waves” in China [7]. The large historical rabies epidemic “waves” in China since 1949 clearly pinpoint dog-mediated rabies as the major source of infection for humans and other animals [8].

Without mass education, strict administration, and forced vaccination, dog rabies in China has spread to ferret badgers (*Melogale moschata*, FBs) in Southeast China, and the virus currently independently circulates in this animal population [9,10]. Severe acute respiratory syndrome coronavirus 2 (SARS-CoV-2) emerged in December 2019 and spread globally, causing a pandemic of respiratory illness designated coronavirus disease 2019 (COVID-19). Following the first outbreak of COVID-19 in China, wildlife has become a global focus topic. Highly strict legislative and regulatory measures need to be performed to restrict wildlife consumption, trade and domestication. These measures subsequently affect the wildlife population because the prevention and control of lethal zoonoses transmitted by wildlife, which have not been previously focused on, are now being considered. Wildlife rabies may have originated from dogs, and the goal of the elimination of dog-mediated human rabies by 2030 is usually ignored due to the lack of vaccination and surveillance in China [11]. Over 100 humans have died from wildlife-associated rabies since the 1990s, and the numbers have been recorded in China's disease-reporting system; the virus has possibly been transmitted to other wildlife or domestic animals and even back to dogs [12]. Without a coordinated national program for rabies control, the reservoir species for disease transmission in China is increasing, and control is becoming increasingly complicated [11].

## 2 Rabies in dogs

At least 95% of human rabies cases in China are caused by bites from rabid dogs [13]. Dog registration and rabies vaccination are mandatory in China, but the policy is not strictly enforced, and no scientific dog demographic study has shown the precise dog population [4]. China has approximately 80 million dogs, with at least 8 dogs/km^2^, which is almost twice the threshold of 4.5 dogs/km^2^ to support endemic rabies [4,8,14]. Dog vaccination coverage varies from rural regions to wealthy cities and ranges from almost 0% to 90% [3,15]. With the low vaccination coverage in dogs leading to the frequent transmission of RABV, these viruses have acquired genetic diversity. Molecular epidemiological studies have indicated that RABV variants circulating in China are divided into 7 phylogenetic lineages (China I–VII); in contrast to the China II lineage, the China I lineage has experienced rapid geographic expansion and become dominant in rabies transmission even in wildlife hosts [11,16,17].

Dogs are owned as pets in urban regions or as family guards in rural areas and are seldom registered and leashed in China [4,17,18]. Unregistered and free-roaming dogs in rural regions and in cities are rabies surveillance shadow area and the main rabies hosts, because veterinarians must administer vaccines only to registered dogs [8,18]. Rabies control has become polarized, leading to excessive vaccination in registered dogs and neglecting administration in unregistered dogs [18,19]. Although the vaccination coverage rate in several major cities in China is higher (at approximately 90%) than the recommended coverage rate of the World Health Organization (WHO) (at 70%), rabies epidemics in humans and dogs still cannot be eliminated because the rabies host is disregarded (unregistered or stray dogs) [3,20].

At least 10 local and 4 imported brands of canine inactivated vaccines have been approved by the Ministry of Agriculture of China and have sufficient production levels for rabies vaccination of registered dogs [21]. However, unregistered and stray dogs are difficult to catch and inject with inactivated vaccines. Oral vaccines for free-roaming dogs and wildlife remain at the laboratory-research levels for certain technical and official reasons in China. Therefore, mass education and strict administration are optional methods to reduce the unregistered dog population. Regulated and civilized breeding can reduce the population of free-roaming dogs and decrease the frequency of dog-biting accidents [18].

Dog-attack injuries transmit RABVs, threaten physical health and induce psychological stress. With the lack of mass education regarding rabies, many people in cities still fear contracting rabies after PEP and thus apply for assays for vaccine-induced antibody levels or receive excessive vaccinations. Incomplete PEP and not immediately seeking PEP are common in rural China and lead to PEP failure and rabies deaths [22,23]. People must be educated on how to treat dog bites and seek immediate, proper PEP and not employ other unapproved methods. The case of the father who died from rabies after sucking his son's dog-bite wound is a painful lesson for public health professionals and reiterates the need to educate people on how to follow WHO-approved standard procedures [24].

## 3 Rabies in FBs

**Fig. 2:**
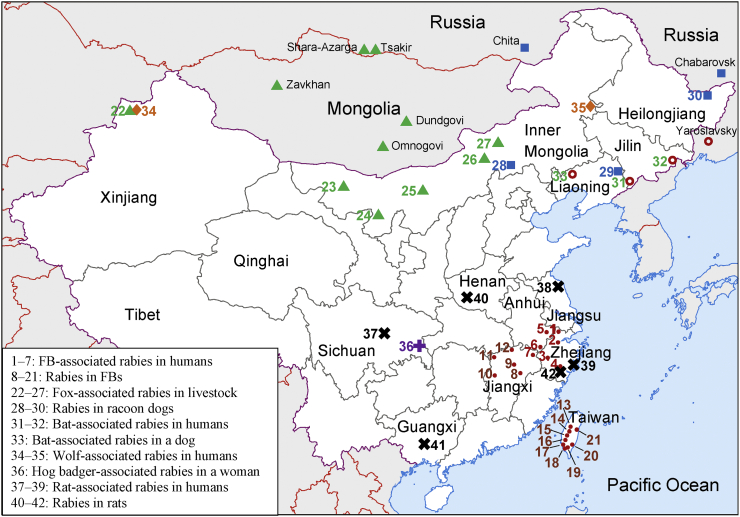
Geographical distribution of wildlife-associated rabies in China and neighboring countries. Red dots indicate the ferret badger-associated cases in Jiangxi, Anhui, Zhejiang, and Taiwan. The green triangles show fox-associated rabies in Xinjiang, Inner Mongolia, and neighboring countries. The blue square represents raccoon dog-associated rabies in Inner Mongolia, Heilongjiang, and neighboring countries. The red circles represent bat-associated cases in Northeast China and the Far East of Russia. The numbers indicate the locations of rabies cases in China, including 1: Huzhou; 2: Hangzhou; 3: Quzhou; 4 and 42: Lishui; 5: Xuancheng; 6: Huangshan; 7: Wuyuan; 8: Fuzhou; 9: Xinjian; 10: Yichun; 11: Xiushui; 12: Hukou; 13: Taichung; 14:Nantou; 15: Yunlin; 16:Chiayi; 17: Tainan; 18: Kaohsiung; 19: Pingtung; 20: Taitung; 21: Hualien; 22 and 34: Tacheng; 23: Alxa Youqi; 24: Alxa Zuoqi; 25: Urat Front Banner; 26: Sonid Youqi; 27: Sonid Zuoqi; 28: Plain and Bordered White Banner; 29: Qingyuan; 30: Tongjiang; 31: Tonghua; 32: Longjing; 33: Fuxin; 35: Jalaite; 36: Mawu; 37: Santai; 38: Binhai; 39: Taizhou; 40: Nanyang; and 41: Nanning. (For interpretation of the references to colour in this figure legend, the reader is referred to the web version of this article.)

FBs have caused at least 87 human deaths since the 1990s [12]. RABV in FBs from Southeast China originates from dogs and independently circulates in the FB population [11]. In the provinces of Jiangxi, Zhejiang, and Anhui, human cases are attributed to FB bites (Fig. 2) [9]. In Taiwan, although rabies in FBs has been reported since 2012, no human cases have been documented [25]. The high percentage (69.6%) of rabies seroconversion in FBs suggests that the occasional exposure of FBs to RABV may cause peripheral abortive infection rather than rabies development in the central nervous system [10,26]. Therefore, rabies pathogenesis and the potential threat of asymptomatic infections in FBs must be further studied [26].

**Fig. 3:**
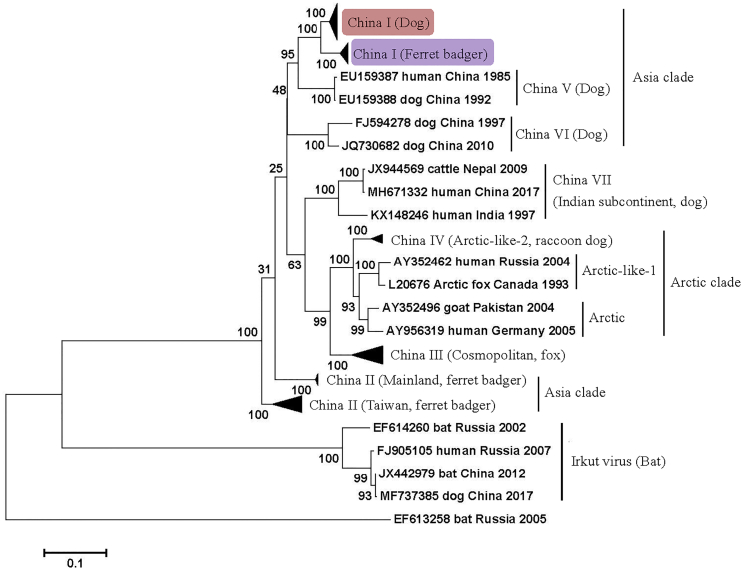
Maximum likelihood phylogenetic tree based on the complete nucleoprotein gene of lyssaviruses isolated from China and neighboring countries.

Most RABV isolates from FBs before 2013 belonged to the China II lineage. In recent decades, the China I lineage has become dominant in rabies transmission in dogs instead of the China II lineage, and since 2014, all FB isolates have been classified under the China I lineage [11,27]. The FB-associated China I sublineage is distinguished from other sublineages in the dog-hosted China I lineage and has possibly become an independent clade in the phylogenetic tree (Fig. 3). This finding suggests the genetic diversity of FB RABVs and the obvious differences in the host range of the lineages from the coexisting dog rabies viruses [11,28]. Therefore, the threat of FB transmission to humans and other animals is complicated and serious because of the influence of dog rabies in China.

To control and prevent FB-associated rabies from the origin, the termination of rabies epidemics in dogs must be prioritized. Apart from targeting dog rabies, to eliminate FB-associated human rabies, the development of oral rabies vaccines for the mass vaccination of FBs must be supported by the appropriate state agencies [29]. In fact, this strategy is not financially prohibitive. Given that FBs frequent rice fields and vegetable gardens as feeding sites, efficient and effective vaccinations can be performed in these areas, thereby avoiding the high cost of the large-scale release of vaccine baits [30].

## 4 Rabies in foxes and raccoon dogs

Wildlife rabies reservoirs include foxes and raccoon dogs in northern China (Xinjiang, Inner Mongolia, Heilongjiang) (Fig. 2). Although rabies transmission by these carnivores to domestic and agricultural animals and the subsequent economic losses have been reported, no human-associated rabies casehas been reported yet [31,32]. However, infected livestock, particularly cattle and camels with possible rabid signs, may present a potential rabies risk to veterinarians and animal handlers [33]. We found that RABVs isolated in foxes and raccoon dogs in China are clustered with isolates from the same target animals in Mongolia or other neighboring countries, suggesting possible cross-border viral transmission [21,31]. Therefore, the regular surveillance of cross-border transmission between China and neighboring countries is necessary for wildlife rabies control.

Inactivated rabies vaccines for large domestic animals, especially cattle, camels, and sheep, have not been developed and produced in China because most large livestock cases have been neglected or treated as occasional accidents, resulting in no demand for vaccines [21]. In China, a single type of vaccine, that is, a canine inactivated vaccine, is currently available, and it can be used for immunizations only via the intramuscular injection route. Although this vaccine can be used to control rabies in domestic animals, the immunization program is not ensured in other animals [21]. Therefore, licensed vaccines for large domestic animals are still required for use in pastoral farms in China.

## 5 Rabies in bats

In 2012, a bat lyssavirus was isolated from an insectivorous greater tube-nosed bat (*Murina leucogaster*) through an active EPI surveillance program in Jilin Province as two reported bat-related human rabies cases. The virus is characterized as Irkut virus (IRKV), which was initially described and isolated in Siberia, Russia (Fig. 2) [34]. This case is the first bat-borne lyssavirus identified in China, and whether it causes any human infection and death remains unknown. IRKV discovery cannot be linked to the two previously reported bat-associated human deaths in Jilin due to a lack of human clinical materials. However, a dead dog that had previously bitten a human was infected with IRKV in 2017, suggesting that additional attention must be given to the trans-species infection of IRKV between bats and dogs or dogs and humans [35]. We found only limited cross-reactivity of IRKV with current rabies biologics in an animal model, suggesting the difficulty of PEP in the prevention of bat-related lyssavirus infections [36,37]. In addition, most recently, a new type of lyssavirus was identified from a dead Japanese house bat carcass in Taipei, Taiwan. Additional information is unavailable on the natural infections of bats with lyssaviruses [38]. Therefore, surveillance for non-RABV lyssaviruses in China is limited, and the threat to public health and veterinary implications must be estimated.

## 6 Rabies in other animals

Rabies in cats has caused approximately 4% of the documented human cases, mostly through cat bites or scratches and always originating from rabid dogs [13,39]. China has no policy guiding cat rabies management and vaccination, although imported cat rabies vaccines are available. Cats do not maintain independent RABV circulation in their population, and the successful control of dog rabies is an effective way to prevent the spread of this disease in cats [8]. Other wildlife in China that have rabies, including rats, wolves, and hog badgers (Fig. 2), are not the original reservoir hosts; rabies in these animals is due to unusual spill-over events from dogs or foxes, and these animals sporadically transmit rabies to humans [12].

## 7 Conclusion

The “one-health” concept has been proposed as the only effective method to eliminate dog-mediated human rabies by 2030; however, although a country may have the appropriate technology, it must be willing to comply with this approach for it to be effective [2]. Such examples are the control of SARS and COVID-19 outbreaks in China [40]. Without strong political commitment, no coordinated national program under the “one-health” concept for rabies control and prevention will be established.

Rabies was well recognized and described in ancient Chinese medicine, and people gained some knowledge or awareness about the disease [7]. However, people must be educated on how to treat dog/animal bites and receive proper PEP on time rather than undergoing unapproved interventions. However, poverty is a problem in rural areas where dog rabies is endemic, and many people cannot afford PEP [7,8]. Governmental subsidies to reduce the cost of PEP and medical insurance coverage of the cost of PEP can alleviate the burden for this population. However, the most cost-effective strategy is to sufficiently administer and vaccinate unregistered dogs [41].

Rabies is preventable by vaccine, and rabies mostly affects the vulnerable population in rural areas with poor education, health infrastructure, and rabies PEP [18,28]. China is fostering a “harmonious society” with fundamental principles of nature, society, and humanity, which are all applicable for rabies control and prevention. Resource limitations or key technical difficulties are not legitimate excuses for China. We need a “champion” who will include government commitment to organize a “harmonized” national program, including intersectoral collaborations, education, dog management, vaccine delivery and mass vaccination, diagnostics, surveillance, and human PEP [14]. The U.S. claimed to be free of dog rabies in 2007 despite the disease burden in raccoons, skunks, foxes, coyotes, jackals, mongoose, and bats [42],which served as the “blueprint” for human rabies elimination to China [2]. China and the U.S. have a similar geographic size and are the top 2 largest economies. To eliminate dog-mediated human rabies by 2030, China must act now before the transmission dynamics in wildlife become increasingly complicated.

## Ethical statement

Not applicable.

## Declaration of Competing Interest

The authors declare that they have no known competing financial interests or personal relationships that could have appeared to influence the work reported in this paper.
